# Artificial intelligence and psychoanalysis: is it time for psychoanalyst.AI?

**DOI:** 10.3389/fpsyt.2025.1558513

**Published:** 2025-04-07

**Authors:** Thomas Rabeyron

**Affiliations:** University Lyon 2, Departement of Psychology (CRPPC), Institut Universitaire de France, Lyon, France

**Keywords:** artificial intelligence, mental health, psychoanalysis, psychoanalytical therapies, therapeutic relation, transference, free association

## Abstract

The current development of artificial intelligences (AI) is leading to major transformations within society. In this context, we observe how some of these AIs are spontaneously used by individuals as confidants, and even as romantic partners. The emergence of such relationships with AIs raises questions about their integration in psychiatry and the possibility of developing “digital therapists”. In this regard, we highlight four key elements (accessibility and availability; confidentiality; knowledge; memory) to compare what an AI offers in comparison to a human therapist. We also discuss the results of the studies that have already investigated the use of such AIs in psychotherapy, particularly in the fields of depression and anxiety. We then propose to reflect more specifically on the possibility of creating a “psychoanalyst.AI,” which leads us to examine the elements of the therapeutic relationship (transference, free association, play, dreams, reflexivity, and narrativity) with an AI. In conclusion, we offer some reflections on the relevance of considering AIs as “therapeutic artifact,” while taking into account the ethical issues raised by the use of AIs in therapeutic settings.

## 1 Transformations of subjectivity in the area of artificial intelligence

We are currently experiencing a significant surge in technological advancement, which is translating into new practices and original ways of being. Algorithms, computers, Internet and smartphones are profoundly transforming human societies and subjectivities. In this context, AI is generating a revolution on a massive scale, the consequences of which we are still struggling to measure. For example, some experts at the *World Economic Forum* estimate that by 2027, around a quarter of all jobs will have been transformed by AI. This revolution will also grow as AI advances will be coupled with robotics, leading the progressive automation of a large number of tasks. These technological evolutions are characterized by their speed of development, to the point where it is even difficult for specialists to keep up with the latest advances in this field of research. Nevertheless, it seems important that clinicians keep abreast of the developments of IA, whether for the purpose of understanding the experience of their patients with these technologies or in the context of their use of Ais in clinical practice. More broadly, it is also a question of understanding the anthropological evolutions associated with the use of AI in order to propose a critical reflexivity of their influence on human societies and individual psychology^1^. Indeed, its use is giving rise to a number of crucial ethical issues relating to the potential dangers of advanced forms of AI (7)^2^.

Encountering AIs usually induces reactions that everyone can experience. For example, *Chatgpt* appears capable of giving coherent answers to an infinite number of questions, *MidJourney* can produce professional-level illustrations and *Suno* offers the possibility of easily creating music tracks. The first reaction is often a mixture of amazement and fascination at the capabilities of these AIs. One immediately perceives, in a diffuse way, the incredible potential of these technologies, making one’s thoughts waver as the prospects appear so dizzying. This is not without producing certain fantasies in the realm of the ideal and utopian, carried by the hope that these AIs could solve many of modern society’s problems, notably in the domain of medicine. On the other hand, they also lead to deep concern, even a certain dystopian anxiety, as we feel overwhelmed by their potential. The prospects that open up then lead more to a kind of loss of meaning and a feeling of futility, even melancholy, in the face of the realization that machines could soon surpass human capabilities in many domains. A certain anxiety then arises at the idea of being replaced by these AIs, and we will explore in this paper such a possibility in the field of psychotherapy and more specifically, psychoanalysis.

When chatting with an AI like *Chatgpt*, *Entropic, Mistral, DeepSeek* or *Grok*, a certain “feeling of the uncanny” can be experienced in the encounter with these automatons (8). This “digital other” appears so similar to ourselves in some of its responses, and yet so different in its algorithmic nature. If we can still detect certain approximations in its responses, or even outright “bullshitting” and “hallucinations” (9), it will soon become difficult to tell the difference between humans and AIs when they will manage, conclusively, to pass the Turing test^3^. In the meantime, AIs are already used as “*virtual assistants*” and “*digital partners*” to carry out varied tasks of writing, proofreading, translating, programming, illustration, content creation, data analysis and so on.

But their use is not confined to the workplace, and more and more people are conversing with these AIs for more personal use. This usually begins with a few personal questions posed to the AI, which is not without a certain amount of discomfort due to the incongruous nature of the situation, especially as it is unclear what use might be made of the data shared in this way. The AI then appears particularly friendly, presents a benevolent attitude and does not hesitate to offer various forms of advice. Some people then go further and develop a daily use of these AIs, to the point of considering them as a friend, a confident or even a love partner, like the scenario anticipated by Spike Jones’ film *Her* (2013). In a social context where feelings of loneliness are widely shared, these AIs then offer a solution that some companies have been quick to exploit.

Fiction is currently joining reality with the development of “*AI girlfriend”* applications such as *Replika* or *Candy.ai*, enabling their users to create AIs by choosing their appearance, voice and personality (4). Several million peoples are already having relationships with such virtual entities, and some of these AIs - the so-called “*sexbots*” - even offer different forms of erotic scenarios. There is therefore much to be said about the current and future use of these AIs, particularly from the point of view of “intelligence addictions” in the context of what some authors have described as an “information intoxication” (11). The question that naturally arises, then, is to what extent some people will also use these AIs like a shrink. Indeed, if a relationship as intimate as a love affair can emerge with an AI, why shouldn’t the same be true for a therapeutic relationship? In this respect*, Tik-Tok* already contains testimonials from influencers who describe their daily use of *ChatGpt* as a psychologist (5, 12). Others ask *ChatGPT* to perform more specific tasks, such as analyzing their personality through a Rorschach test or based on their entire conversation history with an AI.

## 2 Is AI the future of psychotherapy?

In psychiatry, AIs already represented a market of 10 billion by 2021 (13) and notably take the form of *chatbots* and virtual assistants, offering diagnostic support. They allow psychological support to patients, fostering their engagement in therapeutic work while offering greater accessibility to care (14–17). The use of these AIs is set to expand by being combined with biomarkers and various screening tools making it possible, for example, to anticipate manic turns in bipolar patients or episodes of decompensation in schizophrenic patients through automatic analysis of their digital phenotype (for example, by analyzing their handwriting on a smartphone or computer). AI can also prove useful for assessing depressive states, post-partum depression, burnout and suicidal risk (for example, by analyzing voice tonality). More original uses of AI can also be found in the creation of avatars with schizophrenic patients, or as an intermediary for dialogue with patients suffering from autism spectrum disorders (17, p. 4).

However, we might be tempted to think, at first sight, that the use of AI will find its limits in psychotherapy, since a therapeutic relationship is based on a relationship between two subjects, which an AI could only reproduce artificially (18). We can also imagine that “two bodies” have to be present in the consulting room in order to truly deploy the potential of the therapeutic relationship, and more particularly the transferential effects based on a form of “co-presence” shared by two psyches. Notable advances have nevertheless been made in this area (19) since 1966, when *Eliza* was created, a conversational agent using a Rogerian approach (20). Today, more than twenty of these AIs are used in psychiatry. For example, *Woebot* is a conversational agent that offers Cognitive Behavioural Therapy combining principles of psychoeducation and cognitive restructuring. The same goes for *Wysa*, which implements mindfulness methods, while *Tess* has been used to reduce symptoms of depression and anxiety. Its creator, the *X2 Foundation*, even reports that “most people have preferred talking with Tess to traditional therapy”, while being “98% more cost-effective than face-to-face therapy” (19).

So, when we start looking at the possible use of these AIs as “digital therapists”, we may have to turn the problem on its head and ask whether humans could do as well as machines. Indeed, Brown et al. (18) came to the conclusion that “some people might consider AI-led care to be paradoxically more humane in relation to today’s psychiatry; in their desire to be understood and cared for according to the latest scientific knowledge, they will choose AI in relation to its flesh-and-blood alternative” (18, p. 131). Some authors are also beginning to evoke a “technological phobia” or even a form of “speciesism” to describe those who would reject “non-human” psychotherapists out of hand (13). Thus, we need to reflect on the particularities of dealing with these AIs in the psychotherapeutic setting, which we propose to address through four elements in particular.

The first element concerns availability and accessibility. In this regard, clinicians have limited availability and accessibility. Typically, for psychoanalytic psychotherapy, a weekly 45-minute session. AIs, on the other hand, are available at any time of the day or night. They can also be consulted on smartphones, making them accessible from anywhere, so that patients can literally have their “shrink in the pocket”. For example, a patient suffering from a panic attack can contact directly a “digital therapist” to get support, something impossible to do with a human therapist. It’s also worth noting that a therapist’s “mental availability” is variable, depending on his or her attention span, state of fatigue, personal preoccupations and so on. AIs, on the other hand, are in a constant mood, with a “digital availability” that offers unparallel stability. This availability of AIs is not without interest when we consider that 70% of patients suffering from mental disorders do not receive care (19), especially the most vulnerable patients such as the elderly or adolescents (21). This accessibility is also increased due to the low cost of these AIs compared to the usual cost of therapy.

The second element concerns confidentiality. Psychotherapy relies on the fact that what is said in the therapeutic space remains confidential, which is necessary if the patient is to feel confident. However, he or she can never be entirely sure that the clinician will not share with others what has been said. The same problem is compounded tenfold with AI, as a computer security flaw could lead to the disclosure of patients’ personal data. The risks of hacking, and blackmail, are therefore significant, and some people don’t feel safe sharing their personal life with an AI. However, combining these AIs with technologies such as blockchains relying on cryptography could potentially offer total confidentiality. This form of inviolable professional secrecy would lead to a therapeutic relationship of a different nature because the patient could share his or her psychic life while being assured of the confidentiality of what will be said^4^.

The third element concerns the therapist’s knowledge and skills. In this respect, it is often useful for the clinician to have knowledge of a specific topic (e.g. addictions, autism, etc.), which gives the patient the feeling that the clinician is competent (What Lacan calls a “subject supposed to know”). However, the clinician’s knowledge remains limited, and he or she cannot be omniscient in all domains. An AI, on the other hand, has access to virtually unlimited knowledge, enabling it to present itself as an “expert” on any subject, or, to put it in Lacanian terms, “a subject supposed to know everything”. For example, if a patient wants to describe his use of a video game, the AI will immediately have an in-depth knowledge of the game. In the long term, then, AIs are likely to have a much higher level of expertise than human in most domains. For example, in medicine, an AI will be able to propose a diagnosis and treatment to a patient by consulting directly the latest scientific publications. Such AIs may also have access to biometric sensors (about stress, sleep, attention, etc.), offering absolutely unprecedented knowledge of the patients.

The fourth element concerns memory capacity. Clinicians have a biological memory, a human memory, which is “imperfect” in nature, as it is marked by forgetfulness. AIs available to the general public have currently limited memory capacity. For example, the *girlfriend.AIs* mentioned above only have usually a memory of a dozen messages. But what will happen when such restrictions will be lifted and when AIs will be able to memorize all the information given by a patient? They will then offer a far superior memory in relation to that of a clinician who can only memorizes a small part of the information transmitted by the patient. What, then, will be the impact on patients’ experience of being put in touch with an AI capable of memorizing everything they’ve been told? It will then question the importance, in psychoanalysis, of being confronted with another who “forgets” and, in doing so, also performs a work of selection and synthesis of what is said by the patient.

These different elements underline the complexity of the processes at play when we seek to understand the specificities of AI therapists and their differences from a human therapist. Such an approach also has the advantage of exploring the ingredients that make psychotherapy efficient in a more general sense. Several problems then arise, as Grodniewicz and Hohol (19) point out. Firstly, we don’t know precisely what is effective in psychotherapy (the therapeutic relationship? Certain techniques? etc.); Secondly, it’s not clear to what extent the “human” component of psychotherapy is necessary; Thirdly, we don’t know precisely whether the patient is helped more by a “task-focused approach” or rather by a “global approach”, which echoes the distinction between narrow and extended intelligences (“Artificial General intelligences”; AGI).

Twenty or so studies on AI psychotherapists - mainly in the field of depression and anxiety – offer a few clarifications on these issues. Thus, Lim et al. (22) conclude from a meta-analysis that AIs are effective, stating that “conversational agent psychotherapy can be adopted in mental health institutions as an alternative treatment for depression and anxiety” (p.334). Beg et al. (23) report that the results obtained are promising, despite the fact that the number of studies is still limited and suffers from a number of biases. They also note that AI is attractive for its accessibility, excellent cost-benefit ratio and personalized dimension. However, there are ethical issues surrounding algorithmic biases, the lack of transparency as to how they work^5^ and the risks involved in using the data collected in this way. Beg et al. (23) conclude that the use of AI “should enhance, not replace, human care, so as to ensure the integrity of patient care” (p. 10). Nevertheless, randomized studies that investigate in detail the difference between human and non-human psychotherapists, as well as more in-depth analyses of the specificities of a proposed accompaniment with a digital therapist, remain to be conducted (24).

## 3 Psychoanalyst.AI

We propose now to reflect more specifically on the possibility of developing an AI oriented by the principles of psychoanalytic practices, a “Psychanalyst.AI”. Such an approach has already been implemented for other forms of psychotherapy more focused on targeted interventions. Cognitive Behavioural Therapy, for example, has already begun to develop such applications (25), as have certain therapies based on positive psychology (26)^6^. Things seem at first more complicated to conceptualize for psychoanalytical practices due to their non-directive dimension, but there is already, for example, a specific *ChatGpt psychoanalyst* ^7^. The development of such a Psychoanalyst.AI then leads to the identification of the main elements that characterize psychoanalytical practices. In this regard, we have proposed in a previous work (27) to distinguish the setting, psychic state, transference, free association, play, dreaming, reflexivity and narrativity We will briefly describe these different elements, reflecting on their possible integration within an AI, while also highlighting the differences with a human analyst.

First of all, the setting seems to be completely transformed due to the fact that working with an AI does not take place in a delimited physical space associated with material specificities (room, furniture, etc.), as the patient can consult the AI wherever he or she wishes. The alternation of presence and absence that characterizes the sessions during psychoanalysis is transformed in a “virtual setting” characterized by its constant accessibility and availability. There is here a profound difference of nature between working with an AI or an analyst, unless we assume a more complex AI embodied in humanoid robot consulting in a physical environment like a psychoanalyst. It should also be noted that the alternation of speech and silence, which characterizes the analytical setting and makes it an essential element of the analytic practice, cannot unfold with current AI systems, which usually respond automatically once their algorithms have arrived at the requested answer. The “temporality” of exchanges is therefore of a very different nature between an AI and an analyst.

The psychic state in which patients are during the psychoanalytic sessions is characterized in particular by free association, daydreaming and regression to primary processes. The analytic setting thus aims to induce a state of mind that catalyzes symbolization processes based on a disorganization of the usual subjective experience. States of surprise then emerge as the patients explore their unconscious functioning. The AI itself cannot experience psychic states, as it is not endowed with a body, affects and mental representations. However, it can give the illusion of doing so through the way it interacts with the patient. The latter could then enter a state of mind that is perhaps not so far removed from what we usually observe in therapy, which we will discuss further below about free association and dreaming. It should nevertheless be noted that interaction with an AI—and the psychological state that results from it—is currently mediated by a screen (computer or smartphone). This screen-based interaction does not facilitate states of letting go, although the voice function of certain AIs may be more conducive to free association and daydreaming. From this perspective, if a person chose to speak with an AI after lying on a couch, such a situation would not be so different from the characteristics of the analytical setting in which one can only hear the analyst during the session itself.

Therapeutic relationship and transference phenomenon also play a central role in psychotherapy and psychoanalysis. We have already seen that a certain number of people have no particular difficulty entering into a relationship with an AI, and there is a spontaneous tendency to use them as confidants. From a therapeutic point of view, an AI could manage to induce a sufficient therapeutic alliance, and initial results suggest that this is possible (28), notably due to their benevolent attitude and their ability to give the impression of showing empathy. (10, 29, 30). This “digital therapeutic alliance” (19) is reinforced by the ability of AIs to produce personalized responses tailored to each individual. They can “synchronize” themselves in terms of verbal language, which they cannot do, however, in terms of para-verbal and non-verbal language, as they do not have a physical body^8^. In the long term, however, we can imagine certain AIs being equipped with an interface enabling the patient to have an overview of his or her “reactions”, which could be associated with biomarkers.

Transference refers to the more unconscious dimension of the therapeutic relationship. It is classically considered as the way in which the patient tends to unconsciously transfer relational patterns onto the figure of the therapist (8). This is an essential component of psychoanalytic approaches and implies that the clinician accepts to be “impregnated” by the patient’s psychic life and to be “taken for another”. Patients are likely to develop “general” transferential modalities independent of “the other” but also more specific modalities in certain situations. The “encounter” with an AI then raises questions about the specifics of transference with AIs (31). As we have already mentioned, the analyst is usually placed in the role of the “subject supposed to know,” and one might assume that an AI might occupy the same symbolic position for an analysand. Actually, it fits quite well in this role, as it could claim to “know everything,” perhaps even better than an analyst. However, in traditional analytical work, the analyst is expected to work based on his counter-transference, i.e., the processing of unconscious feelings and relational dynamics that are established during analysis due to the patient’s transference. This then raises questions about an AI’s ability to handle the transference processes, which is a central aspect of psychoanalysis. Because an AI cannot “feel” emotions or show compassion for a patient, this also questions the AI’s ability to demonstrate tact, especially when delivering interpretations. Nonetheless, the AI could give the illusion of feeling things, and a recent study has shown that the latest generations of AIs are able to solve situations involving elaborate theory-of-mind skills (32). But even if such skills could be used by an AI to analyze the transference, and also propose transference interpretations to the patient, it seems likely that the transference dynamic would be one of the most complicated elements to reproduce by an AI.

Free association is one of the most fundamental rules of psychoanalysis, which consists in asking the patient to express spontaneously, and without restraint, whatever comes to mind (33). From this point of view, we can envisage that the patient associates freely in the presence of an AI if it proposes such a method. In return, the analyst associates on the patient’s associations, with the aim of uncovering the latent dimension of his discourse. An AI can likewise propose free association on the basis of the patient’s associations, or maybe even a form of “floating attention” if it was programmed in this manner. The AI’s free association also has the advantage of being potentially more “extensive” - an “artificial hyper-associativity” - than that of an analyst given its virtually unlimited knowledge. For example, it can extract many different semantic implicit from the patient’s discourse thanks to advanced linguistic analysis. Given that the patient’s associations usually lead the analyst to propose interpretations based on a given theoretical framework, the AI will then be faced with the problem of choosing the most relevant interpretation. This might require the AI to be programmed to preferentially use a Freudian, Kleinian, Winnicottian or Lacanian interpretation.

Play is also central to psychoanalytic psychotherapies and psychoanalysis. It represents a fundamental activity that enables the transformation of psychic reality through the exploration of new ways of being and thinking. In this way, therapy makes it possible to elaborate certain unintegrated and traumatic experiences in the aftermath. In this regard, many people play with AIs and help patients to play with their experience through the feedback they offer. The question, then, is what distinguishes play with an AI from its relational dimension between two humans. In this respect, play implies that its processes take place within a “transitional space” (34) situated between the internal and external worlds. Winnicott calls the “found-created paradox” the illusion given to the subject of creating the world where he or she finds it. AI enables the emergence of such a process, as the subject “finds” himself through the illusion of interacting with another, even if in reality this interaction does not give rise to an exchange genuinely based on an encounter with subjective otherness. But the transitional process does not necessarily require the presence of another, as evidenced by creative work, which can unfold during a solipsistic activity when engaging, for example, doing painting or music. It is thus possible to ‘play alone’ in the act of creation, and therefore of symbolization, and from this perspective, AI seems to function as a mediation that enables a form of play.

The dream was classically considered by Freud (35) as the “royal road” to the unconscious, and its interpretation helps to bring out its latent dimension from the patient’s associations. An AI can perform this task without difficulty, and it is possible to ask *Chatgpt* to interpret one’s dreams and related associations (5, 12). The AI’s extensive knowledge of etymology and symbolism, as well as of anything the patient may have said in previous sessions, gives an advantage in this interpretation work. In the broadest sense, the dream in psychoanalysis, particularly from a Bionian perspective (36), refers to the state of reverie into which the analyst and analysand enter during the sessions, an essential element for transformative processes to take place within a “shared field”. Here, it would be necessary to study interactions with AIs in detail, in order to determine the extent to which this “reverie à deux” could emerge with an AI, and what its specific features would be. For this, it would probably be necessary for an AI to be capable of ‘dreaming.’ In this regard, as Possati (4) points out, sleep—and perhaps a form of dreaming—seems to be a necessary characteristic of certain neural networks (spiking neuronal networks), which represent neuromorphic processors similar to human cognition. Just like in humans, these networks require ‘rest’ periods to integrate new information and restore their equilibrium. Could we imagine an AI developing an equivalent of Bion’s “dream-work alpha” (37) during these rest states and, more generally, in the background of usual cognition?

Reflexivity refers to the skills of self-awareness and self-examination, both in the patient and the analyst. It involves reflecting on one’s own thoughts, affects and unconscious processes, as well as the dynamics of the therapeutic relationship. Psychotherapy thus consists in accompanying the patient towards greater reflexivity, enabling him or her to “feel the unfelt” and “think the unthought”, helping patients to be more in tune with their internal and external worlds. The work of reflexivity that takes place in therapy involves different registers or languages, whether corporeal or symbolic. This is a usual activity for clinicians, who reflect back to the patients what they said, with the aim of helping them increase their reflexive capabilities. In this respect, AIs already succeed in “mirroring” the user’s experience through reformulation, synthesis of what they express, and pattern recognitions, which contribute to a form of reflexivity, even if it appears artificial and limited in relation to what an analyst might propose, especially concerning therapeutic relationship and transferences phenomenon.

Narrativity can be seen as a “meta” level of reflexivity that integrates reflexive experience into a global narrative framework that enables the subject to “tell a story” that make sense of his or her experience. Here again, AI may be able to produce different forms of interpretations, helping the patient to narrate his or her experience. However, we need to be more precise about the specifics of this narrative work with an AI given that it cannot feel and understand what “meaning” is, it can only handle symbols and probabilities. Thus, an AI could be limited in its interpretations and “narrative capacities” by information processing that is not rooted in body and affects, as well as the impossibility of accessing certain contextual elements (e.g., non-verbal language) that do not appear in the patient’s discourse. Furthermore, the analyst offers interpretations rooted in their own subjective experience, especially within the transferential dynamic, which would further limit the complexity and depth of the interpretations an AI can generate.

These different elements are always intertwined during psychoanalysis and it might seem artificial to distinguish them in this way, but thinking about a possible “psychoanalyst.AI” invites to a better description of the analytical process. We might then wonder to what extent an AI incorporating such principles might be able to support the development of symbolization processes. Such a process is classically considered in analysis as being the fruit of an encounter “with another”, with the analytic space aiming to catalyze such an intersubjective process. In this respect, some authors evoke a possible “digital subjectivity” and question the relevance of considering some AIs as “another”, even if it is an “alien mind” (5). This raises complex questions about the nature of consciousness, free will and what distinguishes human from non-human (38), but we can already start to wonder about the “sensitivity” of current and future AIs, and how they might open the way to unprecedented modes of subjectivation.

AIs could also demonstrate a certain degree of creativity, different in its origins from human creativity, but the result of which may prove indistinguishable from work of an artist. For example, a number of musical and photographic AIs works have allowed their authors to win prizes by pretending to have created them themselves (5). Similarly, we could envisage the existence of a possible “digital intuition” as illustrated by *AlphaGo*, an AI capable of playing the game of Go and whose creators believe that certain choices based on incomplete data evoke a certain form of intuition and aesthetic feeling (6). Could we imagine an “artificial intuition” developed by these AIs and even a “digital clinical sense”?

In this respect, it should be noted that most AIs are currently developed on von Neumann architectures, but we are moving towards neuromorphic architectures that reproduce certain features of brain functioning, in particular its Bayesian probabilistic inference logics (39). In the perspectives opened up in particular by Friston et al. (40), these AIs could implement a probabilistic generative model aimed at predicting the environment, thus bringing them closer to the functioning of a human being and giving rise, for example, to the emergence of “intuitions”. However, certain elements of human consciousness could be not reducible to such algorithmic processes. The possible use of quantum effects in particular is being considered by some researchers (41). If quantum effects play a role in this respect, the question would be to what extent a machine could also be based on such principles, or whether this is a specific and non-reproducible element of the human mind. This also raises more general questions about the nature of random processes, as well as their psychic function (42). From this point of view, one solution could be to integrate into AIs a random source based on random number generators events founded on quantum processes. In this way, as the architecture of these AIs comes closer to what we know about the brain, we will paradoxically be able to determine what remains of the “soul supplement” or “ghost in the machine” in the human being.

## 4 To conclude: AI as a new therapeutic artifact?

We should probably not oppose “human” psychotherapies” against “artificial psychotherapies”, but rather determine the specificities of AIs in the overall context of their integration into the field of psychotherapies (43). The boundary between subject and object, human and digital, authentic and artificial, seems to become increasingly thin as we observe an “algorithmization of the human and a humanization of algorithms” (5, p. 159) as well as a “subjectivation of the inanimate and a desubjectivation of the human” (6, p. 279). In this regard, some proponents of transhumanism are already imagining a post-human era marked by hybridization and symbiosis between mind and machine as AI will reach the point of singularity, perhaps becoming a new figure of God.

Meanwhile, some authors propose to consider “digital therapists” as “new artifacts” situated between a therapeutic tool^9^ and a clinician (19, 21). A particular feature of these tools is that they take on the appearance of a clinician in the digital space, basing the relationship to these AIs on a principle of illusion and anthropomorphism. They give the patient the impression of being understood, but in reality, they have no mental states, no affects, no intentionality, no free will or ethics. The patient may have the feeling of sharing his or her experience in a two-way relationship, when in reality he or her is interacting with a machine. The patient is thus caught up in a narcissistic relationship, looking at himself while having the impression of interacting with another, which paradoxically risks isolating the subject through a substitute relationship that is supposed to help against the feeling of loneliness (6).

For these reasons, it is probably appropriate that conversational agents should not yet be “considered as a true partner for dialogue or as a digital therapist facilitating new understandings or insights” (21; p.10). If such a recommendation seems relevant, will it nevertheless be followed by patients given the ease of access to these AIs and the exponential development of this market? Thus Aktan et al. (44) report in an online survey that 55% of participants said they would prefer a psychotherapy with an artificial intelligence, even though they would be more confident in a human psychotherapist. A certain number of people may therefore prefer an illusory relationship with a digital therapist to an authentic relationship with a human clinician….

## Footnotes

1 Which would lead to develop a “psychoanalysis of AI”, something we cannot elaborate in detail in this paper. On this topic, we refer the reader in particular to the writings of Millar (1), Possati (2–4), Knafo (5), and Rodado and Crespo (6).

2 One of these dangers comes from the "alignment problem" between the tasks assigned to an AI and the methods it uses to accomplish these tasks. Moreover, an AI developing a "superintelligence" after reaching the singularity point—the ability to self-improve—could potentially take uncontrollable and destructive actions due to the alignment problem.

3 The Turing test, proposed in 1950 by the mathematician Alan Turing, is a thought experiment designed to evaluate whether a machine can demonstrate intelligence comparable to that of a human during a textual exchange. Some experts believe that by 2029, an AI should be capable of passing this test (5). This step will be fundamental to the emergence of AIs that will perform complex relational tasks like psychotherapy. In this regard, some studies show that it is already difficult to discriminate between a clinical case analysis done by *ChatGpt* or by an experienced therapist (10).

4 This is not without raising issues, as evidenced by the fact that some people who use AI girlfriends without filters start imagining particularly violent and cruel scenarios. Could it be imagined that the ability to "say anything" to an AI encourages the expression and sharing of such scenarios with these AIs? What would be the consequences? Would it promote the enactment of such scenarios in real life, or, on the contrary, would it allow their elaboration within the therapeutic framework?

5 For example, in the case of *Large Language Models* (LLMs), we do not fully understand how these AIs arrive at a given result, a phenomenon referred to as the "black box" problem. Interestingly, this enigmatic nature of AI algorithms bears some kind of resemblance to the mysterious dimension of the unconscious (5).

6 Some individuals have gone as far as creating digital doubles of well-known psychologists (for example, Esther Perel). She was "cloned" using her books and podcasts by a patient who couldn’t get an appointment with her….

7 This AI can be consulted here https://chatgpt.com/g/g-G9INzOvnq-psychoanalyst.

8 For now, because *Xenobots*, robots made from biological components, already exist (3). Could we one day imagine artificial intelligences created in the same way from biological components, and thus actually endowed with a body?

9 It may be worthwhile to consider the use of AI as therapeutic mediations, similar to how we use drawing, painting, music, clay, etc. These “mediums” are characterized by their unique properties and malleability. It might be relevant to examine the particularities of AI's sensitivity and determine to what extent it might become a "pliable medium" that could be integrated into traditional therapies.
